# Factors associated with anaemia among women of reproductive age in Ethiopia: Multilevel ordinal logistic regression analysis

**DOI:** 10.1111/mcn.13063

**Published:** 2020-08-05

**Authors:** Lire Lemma Tirore, Afework Mulugeta, Abate Bekele Belachew, Menaseb Gebrehaweria, Abraham Sahilemichael, Desta Erkalo, Rigeat Atsbha

**Affiliations:** ^1^ Department of Public Health, College of Medicine and Health Sciences Wachemo University Hossana Ethiopia; ^2^ Department of Nutrition, School of Public Health, College of Health Sciences Mekelle University Mekelle Ethiopia; ^3^ Department of Biostatistics, School of Public Health, College of Health Sciences Mekelle University Mekelle Ethiopia; ^4^ Department of Public Health, College of Medicine and Health Sciences Adigrat University Adigrat Ethiopia; ^5^ School of Health Sciences University of Brighton Brighton UK; ^6^ Saesie Tsaeda Emba District Health Office Tigray Regional Health Bureau Mekelle Ethiopia

**Keywords:** anaemia, Ethiopia, multilevel ordinal logistic regression, women

## Abstract

Anaemia has prevailed as a mild to severe public health problem in Ethiopian women of reproductive age. Many studies carried out on anaemia have been limited to subnational assessments and subgroups of women. The effects of potential factors thought to affect anaemia and severity levels of anaemia have not been well considered. Therefore, this study identifies individual, household and community level factors associated with anaemia among women of reproductive age in Ethiopia applying multilevel ordinal logistic regression models. Proportional odds assumption was tested by likelihood ratio test. About 35.6% of the variation on anaemia was due to between household and community level differences. Pregnancy (adjusted odds ratio [AOR] = 2.30, 95% confidence interval [CI]: 1.82, 2.91), HIV (AOR = 2.40, 95% CI: 1.76, 3.25), giving birth once (AOR = 1.2, 95% CI: 1.05, 1.40), giving birth more than once (AOR = 1.4, 95% CI: 1.19, 1.71), living with five or more family members (AOR = 1.24, 95% CI: 1.05, 1.47), living in poorest households (AOR = 1.34, 95% CI: 1.2, 1.61) and rural area (AOR = 1.57, 95% CI: 1.28, 1.92) were associated with greater odds of more severe anaemia compared with their respective counter parts. Secondary and above education (AOR = 0.83, 95% CI: 0.70, 0.97) and use of pills, implants or injectable (AOR = 0.67, 95% CI: 0.59, 0.77) were associated with lower odds of more severe anaemia. Anaemia prevention and control programmes need to be strengthened for women living with HIV/AIDS and during pregnancy. Household poverty reduction and social protection services need to be strengthened and integrated in anaemia prevention and management activities in women.

**Key messages**

- Anaemia remains a public health concern in women of Ethiopia.
- Anaemia was found to be influenced by factors evolving at individual, household and community levels.
- There was a sizable heterogeneity in likelihood of anaemia across households and clusters.
- Pregnancy, HIV, giving birth once or more than once, living with five or more family members, living in poorest households and rural area were associated with greater odds of worse anaemia. Secondary and above education and use of pills, implants or injectable were associated with lesser odds of worse anaemia.
- The Ethiopia FMoH should strengthen anaemia prevention and control programmes for vulnerable WRA.

## 1 INTRODUCTION

Anaemia is a global public health problem affecting about one‐third (32.8%) of all women of reproductive age (WRA), 40% of pregnant and 32.5% of nonpregnant women in 2016 (The World Bank, 2018). It is a severe public health problem in many countries of the world. Defined as a condition characterized by a lower than normal concentration of haemoglobin (Hb) (World Health Organization, 2015), anaemia contributes to maternal and perinatal mortality. It doubles the risk of maternal death and accounts for 20% of all maternal deaths (Balarajan, Ramakrishnan, Özaltin, Shankar, & Subramanian, 2011; Black et al., 2008; Daru et al., 2018).

Worldwide, each year, anaemia results in more than 115,000 maternal and 591,000 perinatal deaths (Stoltzfus, Mullany, & Black, 2004). Perinatal anaemia increases the risk of other poor pregnancy outcomes like infection, preterm birth, low birth weight, small size for gestational age and anaemia during infancy (Kozuki, Lee, & Katz, 2012; Rukuni et al., 2016). Impairing cognitive (Haas & Brownlie, 2001) and motor development, it reduces work capacity and productivity (Vanishri, Kumar, & Kiran Singh, 2017). The aggregated effects of such consequences pose a substantial economic burden to individuals and country (Horton & Ross, 2007; Vanishri et al., 2017; World Bank, 2009).

Iron deficiency is the most common underlying cause of anaemia contributing to more than half of all cases anaemia (Hay et al., 2017). Other micronutrient deficiencies (e.g., folate and vitamin B12), acute and chronic diseases (e.g. malaria, hook worm) and inherited blood disorders that affect red blood cells (RBC) production or survival are other common causes of anaemia (Balarajan et al., 2011; Kassebaum et al., 2014). Risk factors of anaemia are many, contextual and complex. These factors operate at different levels of the hierarchy, i.e. individual (e.g., education, infection, reproductive characters, behaviour and nutritional factors), household (wealth, family size and sanitation facility), community (cultural taboos, residence, access to health service and clean water) and at district, regional and national levels (Adamu et al., 2017; Haverkate, Smits, Meijerink, & Van Der Ven, 2014; Kamruzzaman, Rabbani, Saw, Sayem, & Hossain, 2015; Kandala, 2013; Perumal, 2014; Wilunda, Massawe, & Jackson, 2013).

Both the risk and consequences of anaemia are more prominent in low‐ and middle‐income countries (Balarajan et al., 2011). Africa was ranked the second highest in terms of anaemia (38.6%) and severe anaemia (1.8%; World Health Organization, 2015). It is a moderate and severe public health problem in all WRA (38.6%) and pregnant women (54%) of the Sub‐Saharan Africa (SSA), respectively (The World Bank, 2018).

The Ethiopian Federal Ministry of Health (FMoH) has been struggling to prevent anaemia focusing on pregnant women by providing iron (Fe) and folic acid, nutrition education, providing drugs for deworming, promoting sanitation and preventing and treating malaria (Federal Ministry Of Health, 2008). However, in the last 15 years, the trend of anaemia has remained inconsistent. Although the prevalence of all form anaemia decreased from 27% in 2005 (Central Statistical Agency [Ethiopia] and ORC Macro, 2006) to 17% in 2011 (Central Statistical Agency [Ethiopia] and ICF International, 2012), it increased to 23.63% in 2016. The prevalence of anaemia was higher among pregnant, breast feeding, rural women and women who have had six or more births. It was also more prevalent with decreasing educational level and household wealth (Central Statistical Agency (CSA) [Ethiopia] and ICF, 2016).

Recognizing it as a worldwide public health problem, the WHO target is set to reduce anaemia in WRA by 50% in 2025 (World Health Organization, 2014). Maternal nutrition is one of the top priorities of Health Sector Transformation Plans (HSTP). The prevalence of anaemia in WRA is among the outcome indicators of HSTP targets (Federal Ministry of Health, 2015). In settings with limited resource like Ethiopia, up‐to‐date and empirical evidence on severity levels of anaemia and their associated factors is essential to achieve such targets and give priority to those who are at risk of higher levels of anaemia (Neha, Moutish, Ramanan, & Purnima, 2015).

Many studies focusing on anaemia in women have been limited to subnational assessments and subgroups of the women, with most studies being carried out with pregnant women. Therefore, the results of these studies are not generalizable to all WRA. Furthermore, the effects of some potential factors like cooking fuel, khat chewing, alcohol consumption, lactation, community education and smoking have been given little attention and are not well understood. Although factors affecting anaemia are considered to operate in different levels, existing national studies used single level analysis techniques which may lead to incorrect estimation of parameters, standard errors and false conclusion of the association. Also, single level analysis cannot measure the contribution of the household and community clustering to the total variation of anaemia (Diez‐Roux, 2000). To the investigators knowledge, the effect of the household level variability on anaemia has not been examined in the context of Ethiopia. The effect of anaemia differs depending on the severity levels (mild, moderate or severe; Riva et al., 2009). Accounting for the ordered nature of anaemia may reduce bias in parameter estimation, increase precision and power and consider as an efficient use of information available (Hedeker, 2015). Multilevel ordinal logistic regression (OLR) examines the effects of explanatory variables at different levels simultaneously. It produces more accurate estimates of regression coefficients, standard errors, confidence intervals and significance tests compared with single‐level logistic regression. Furthermore, it quantifies within and between groups variation on outcome which might be due to unobserved factors at different levels (Diez‐Roux, 2000).

The aim of this study, therefore, was to identify individual, household and community level factors of anaemia in WRA simultaneously using multilevel OLR analysis technique. Additionally, the study aimed to quantify the amount of unexplained variation in odds of anaemia which is attributable to community and household clustering.

## 2 METHODS AND MATERIALS

### 2.1 Data sources

**FIGURE 1:**
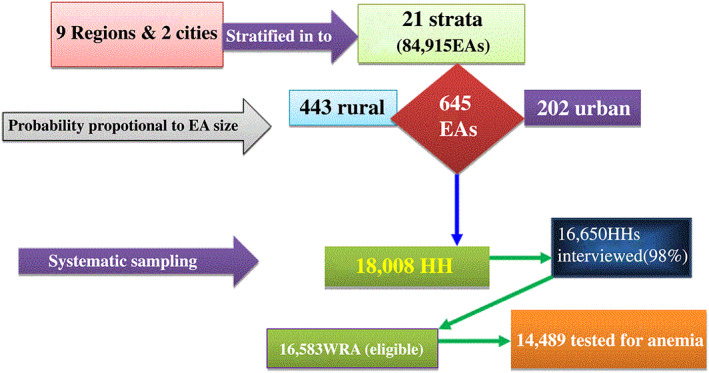
Sample size and sampling procedure for factors associated with anaemia among women of reproductive age in Ethiopia, 2018: Data from 2016 Ethiopia Demographic and Health Survey (EDHS)

This study used 2016 Ethiopia Demographic and Health Survey (EDHS) data set collected from the nine regions and two administrative cities of Ethiopia. The 2016 EDHS is the fourth and the most recent nationally representative survey conducted with the main objective of providing timely and reliable data on health and demographic outcomes (Central Statistical Agency [CSA; Ethiopia] and ICF, 2016). Stratified two‐stage sampling technique was used to select enumeration areas (EAs) and households. An EA is a geographic area covering on average 181 households (HHs). The 2007 Ethiopia Population and Housing Census (PHC) was used as a sampling frame to select EAs. In the first stage, 645 EAs (202 in urban and 443 in rural) were selected with probability proportional to EA size. The EA size is the number of residential households in the EA as determined in the 2007 PHC. And those EAs with more households have higher probability of being selected. In the second stage, 18,008 HHs were selected by systematic sampling technique, with average of 28 HHs per EAs. All WRA in the selected HH were eligible for anaemia testing (Figure 1; Central Statistical Agency [CSA; Ethiopia] and ICF, 2016).

After obtaining permission from the Inner City Fund (ICF) International; individual, household and HIV data sets were downloaded from the DHS website (http://dhsprogram.com).

Details on sampling technique, sample size, data collection tools, data quality control and ethical concerns are available in 2016 EDHS report (Central Statistical Agency [CSA; Ethiopia] and ICF, 2016).

### 2.2 Analytic samples

All WRA who had data on anaemia status were included in this study. A total of 14,489 WRA who were tested for anaemia were included in the analysis.

### 2.3 Anaemia

Anaemia is an ordered categorical variable categorized as none, mild, moderate and severe anaemia based on Hb level. Blood samples were taken from a finger prick of the voluntarily consented women and collected in a micro cuvette. Hb analysis was carried out on‐site using a battery‐operated portable HemoCue analyser (Central Statistical Agency [CSA; Ethiopia] and ICF, 2016). Hb levels were adjusted for pregnancy because during pregnancy the increase in maternal blood volume and the iron needs of the fetus decrease the blood Hb level (World Health Organization, 1989). It was also adjusted for smoking and altitude. The World Health Organization (WHO) Hb cut off points for diagnosis of anaemia are given in Table 1 (World Health Organization, 2001).

**TABLE 1:** The WHO Hb cut off points for diagnosis of anaemia

Anaemia status	Hb cutoffs for pregnant women (g/dl)	Hb cutoffs for NP‐NL (g/dl)
Non	>11	>12
Mild	10.0–10.9	10.0–11.9
Moderate	7.0–9.9	7.0–9.9
Severe	<7.0	<7.0

Abbreviations: g/dl, gram/deciliter; Hb, haemoglobin; NP‐NL, neither pregnant nor lactating; WHO, World Health Organization.

### 2.4 Predictors of anaemia

After reviewing recent literature, potential risk factors of anaemia were extracted from the data set. Due to the hierarchical nature of the 2016 EDHS data, the extracted variables were classified as individual, household and community level variables. Individual level variables were characteristics of the women which were specific to each woman (Table 2).

**TABLE 2:** Individual level variables extracted from EDHS 2016 data set for studying factors associated with anaemia

Variable	Description	Category
Age	The age of the woman in years at the time of the survey	0. 15–24 1. 25–34 2. 35–49
Women education	The highest educational level attained at the time of survey	0. No formal education 1. Primary 2. Secondary and above
Religion	The religion of women during at a time of survey	0. Protestant 1. Orthodox 2. Muslim 3. Other
Marital status	Marital status of women at a time of survey	0. Not living with husband 1. Living with husband
Mass media exposure	Listening to the radio or watching TV at least once a week	0. Yes 1. No
Alcohol consumption	Frequency of alcohol consumed within 1 year before survey	0. None 1. Less than once/week 2. At least once/week 3. More than once/week
Khat	Khat chewing habit of respondents within the last month before survey	0. Yes 1. No
Maternity status	Women were asked whether they were pregnant, lactating or neither at a time of survey	0. Pregnant 1. Lactating 2. Neither pregnant nor lactating
History of abortion	Women were asked whether they had history of abortion	0. Yes 1. No
Contraceptive method	Any method women were using at a time of survey	0. None 1. Pill/injectables/implants 0. IUD 1. Nonhormonal
Number of birth in last 5 years	The number of births a women gave in a past 5 years before survey	0. No 1. One child 2. More than one children
Use of deworming drug	Women were asked if they had used a deworming drug during their recent pregnancy.	0. Yes 1. No
Iron supplementation	Women were asked if they had used iron supplements during their recent pregnancy	0. Yes 1. No
Nutritional counselling	Women were asked whether they were counselled or not during their recent pregnancy.	0. Yes 1. No
HIV status	HIV status of women at a time of survey	0. Negative 1. Positive

Abbreviation: EDHS, Ethiopia Demographic and Health Survey; IUD, intrauterine device.

Household level variables are household level characteristics which are common for all women living in the same household and include variables described in Table 3.

**TABLE 3:** Household level variables extracted from Ethiopia Demographic and Health Survey 2016 data set for studying factors associated with anaemia

Variable	Description	Category
Wealth index	Scores were given to households based on the number and kinds of consumer goods they owned.	0. Poorest 1. Poor 2. Middle 3. Rich 4. Richest
Family size	The number of household members with which the woman was living.	0. ≤2 1. 3 and 4 2. ≥5 persons
Cooking fuel	Categorized as: cleaner fuel (electricity, liquefied petroleum gas [LPG], natural gas, biogas and kerosene); or solid fuel (coal/lignite, charcoal, wood, straw/shrubs/grass, agricultural crop and animal dung).	0. Cleaner fuel 1. Solid fuel
Toilet facility	Categorized as: improved (any nonshared toilet of the following types: flush/pour flush toilets to piped sewer systems, septic tanks and pit latrines, ventilated improved pit [VIP] latrines, pit latrines with slabs and composting toilets); or unimproved (shared toilet, flush/pour flush not to sewer/septic tank/pit latrine, pit latrine without slab/open pit, hanging toilet/hanging latrine and other).	0. Improved 1. Unimproved
Drinking water source	Categorized as improved sources (piped water, public taps, stand pipes, tube wells, boreholes, protected dug wells and springs and rainwater); or unimproved sources (unprotected dug well and spring, tanker truck/cart with small tank and surface water).	0. Improved sources 1. Unimproved sources

Community level variables were characteristics which are common for all women residing in the same community (cluster) and include place of residence, region, community (cluster) women education, community poverty, community women unemployment and community mass media exposure. Variables like community women education, community poverty, community women unemployment and community mass media exposure were generated by aggregating individual characteristics within the cluster. The generated variables were further categorized as low or high based on the national median values of the generated variables. These variables are measured as shown in Table 4.

**TABLE 4:** Community level variables extracted from Ethiopia Demographic and Health Survey 2016 data set for studying factors associated with anaemia

Variable	Description	Category
Place of residence	The place where the women residing at a time of survey.	0. Urban 1. Rural
Region	The region of the women at time of survey.	0. Tigray 1. Afar 2. Amhara 3. Oromia 4. Somali 5. Benishangul Gumuz 6. SNNPR 7. Gambela 8. Harari 9. Addis Ababa 10. Dire Dawa
Community poverty	Defined as the proportion of women who resided in poor or poorest households within the cluster. The aggregate of individual households with poorest or poor wealth index can show overall poverty of the cluster. It was categorized as high if clusters had more than or equal to national median proportion (30%) of poorest or poor households or low otherwise.	0. Low 1. High
Community unemployment	Defined as the proportion of women who were not currently working within the cluster. The aggregate of individual women without work can show overall unemployment condition of the cluster. It was categorized as high if cluster has more than or equal to national median proportion (50%) of women without work or low otherwise.	0. Low 1. High
Community mass media exposure	Defined as the proportion of women who had mass media exposure within the cluster. The aggregate of individual women with mass media exposure can show overall mass media exposure of the cluster. It was categorized as high if cluster has more than or equal to national median proportion (13.8%) of women with mass media exposure or low otherwise.	0. High 1. Low
Community women education	Defined as the proportion of women who attended primary/secondary/higher education within the cluster. The aggregate of individual woman's primary/secondary/higher educational level can show overall educational attainment of the women in the cluster. It was categorized as high if clusters with more than or equal to national median proportion (7.7%) of primary/secondary/higher education or low otherwise .	0. High 1. Low

### 2.5 Statistical analysis

#### 2.5.1 Multilevel OLR analysis

Due to hierarchical nature of the 2016 EDHS data where individuals are nested within households and households are in turn nested within clusters, multilevel (three‐level) OLR was used. Ignoring hierarchical nature and use of single‐level analysis could result in biased estimation of parameters and standard errors. Furthermore, the assumption of independent observation in ordinary logistic regression does not hold true in hierarchical data. Multilevel analysis handles these limitations by examining simultaneously the effects of explanatory variables at different levels (Diez‐Roux, 2000).

OLR is a well‐suited technique to this study because of the ordered nature of outcome variable (none, mild, moderate and severe anaemia; Hedeker, 2015).

Stata software version 14 was used for analysis of the data. A *P*‐value ≤ 0.25 in bivariate analysis was used to consider candidate variables for multivariable analysis (Stoltzfus, 2011). In a multivariable analysis, a *P* value < 0.05 was used to identify variables significantly associated with anaemia. Adjusted odds ratios with 95% confidence intervals were estimated and interpreted (Raman & Hedeker, 2005). The proportion of variations in odds of anaemia between households and communities was expressed using variance partition coefficients (VPC). The VPC measures the proportion of outcome (anaemia) variation unexplained by the predictor variables that lies at each level of the model hierarchy. It measures the relative importance of clusters, households and individual (women) as sources of variation on anaemia status (Leckie & French, 2013).

### 2.6 Model specifications

#### 2.6.1 Multilevel (three‐level) OLR model

The mixed‐effects OLR (proportional odds) model can be written in terms of the cumulative logits as below in the box:

Log $\left[\frac{\text{Pijkc}}{1-\text{Pijkc}}\right]$ = ᵧ*c* − (*x*_*ijk*_ *β* + *u*_*ij*_ + *u*_*i*_)

*P*_*ijkc*_—is accumulative probability of being at ‘c’ category of anaemia for *k*th individual in *j*th household and *i*th cluster.

ᵧ*c*—is a model threshold or intercept for C‐1 level of anaemia, and it is a fixed parameter.

It represents the cumulative logits of being at or below C‐1 level of anaemia when the covariates and random effects equal to zero. It is strictly increasing (i.e., γ1 < γ2 < · · · < γC − 1).

*C* = number of categories of anaemia which equals to 4.

*β*—is a coefficient (fixed effect of explanatory variable).

*X*_*ijk*_—is a covariate vector for *k*th individual in *j*th household and *i*th cluster.

*u*_*ij*_—is level‐2 (household) random effect, and it is assumed to be normally distributed with variance σ^2^(v2).

u_*i*_—is level‐3 (cluster) random effect, and it is assumed to be normally distributed with variance σ^2^(v3)_._

(Raman & Hedeker, 2005).

Violation of the proportional odds assumption is common. In such occasions, a model which relaxes the assumption is nonproportional or partial‐proportional odds model in which covariates are allowed to have different effects on the C *−* 1 cumulative logits. It is given as below in the box:

Log $\left[\frac{\text{Pijkc}}{1-\text{Pijkc}}\right]$ = ᵧ*c* − (*x*_*ijk*_ *β* + *u*_*ijk*_ *α*_c_ + *u*_*ij*_ + *u*_*i*_)

*u*_*ijk*_—is a covariate vector for set of variables for which proportional odds is not assumed.

*α*_c_—is a vector of regression coefficients associated with these covariates for C‐1 levels of outcome.

Because *α*_c_ carries the c subscript, the effects of these covariates are allowed to vary across the C *−* 1 cumulative logits.

(Raman & Hedeker, 2005)

#### 2.6.2 Random effects

Both household and cluster random effects variance was expressed in terms of VPC.

VPC_(3)_ is a proportion of total variation on anaemia attributable to cluster random effect.

It is given as in the box:

VPC_(3)_ = $\frac{\sigma^{2}\left(v3\right)}{\sigma^{2}\left(v3\right)+\sigma^{2}\left(v2\right)+\pi^{2}/3}$, where π^2^/3 is individual level variance which equals to 3.29.

σ^2^(ν3)—is cluster (level‐3) random effect variance.

σ^2^(ν2)—is household (level‐2) random effect variance.

VPC for level‐2 and 3 clustering effects (VPC_(2 + 3)_) is a proportion of total variation on anaemia attributable to both household and cluster level random effect. It is given as below (Leckie & French, 2013).

VPC_(2 + 3)_ = $\frac{\sigma^{2}\left(v2\right)+\sigma^{2}\left(v3\right)}{\sigma^{2}\left(v3\right)+\sigma^{2}\left(v2\right)+\pi^{2}/3}$.VPC_(2)_ is a proportion of total variation on anaemia attributable to household level random effect.

It is given as: VPC_(2)_ = $\frac{\sigma^{2}\left(v2\right)}{\sigma^{2}\left(v3\right)+\sigma^{2}\left(v2\right)+\pi^{2}/3}.$

The explained variances at cluster and household level were quantified by proportional change in variance (PCV; Merlo, Yang, Chaix, Lynch, & RÅstam, 2005).

#### 2.6.3 Proportional odds assumption

The proportional odds assumption states that the effects of all covariates are constant across categories of outcome variable. After fitting both proportional and nonproportional odds models, the proportional odds assumption was tested using likelihood ratio test. It tests the null hypothesis that there is no difference in the effects of explanatory variables across the levels of anaemia. The *P* value ≥ 0.05 is desirable to retain null hypothesis (Bauer & Sterba, 2011). The likelihood ratio test supported the nonproportional odds assumption. Furthermore, each variable in the model was tested to identify the variables for which the proportional odds assumption was violated.

#### 2.6.4 Model fit statistics

An Akaike information criterion (AIC) was used to select the final model which fits the data best compared with other fitted models. The AIC of the all models were compared, and the model with the lowest AIC was considered as the best model fits the data (Hox, Moerbeek, & Van De Schoot, 2010; Table 6 **)**.

#### 2.6.5 Significance test of random effects

For a substantial number of clusters and households, the 95% confidence intervals of random intercepts do not overlap zero. This implied that the random effects of the many households and clusters on anaemia were significantly different from zero (above or below zero; Leckie & French, 2013).

#### 2.6.6 Model adequacy (normality of random effects distribution)

The normality assumptions were tested graphically using quantile‐quantile plots (Leckie & French, 2013). The result suggested that cluster and household random effects were approximately normally distributed, respectively. This implied that the final model is appropriate for predicting the outcome variable and describing the data at hand (adequate).

### 2.7 Ethical considerations

The permission for access to the data was obtained from ICF International by registering and stating the objective of the study. The data set has no individual names or house hold addresses. The data were used for the registered research topic only and were not shared to another person.

## 3 RESULTS

Table 5 shows individual, household and community level characteristics of the study population. The mean ± standard deviation (*SD*) age of the respondents was 28 ± 9 years. More than half (62.20%) and 1,055 (7.30%) of the women were neither pregnant nor lactating and pregnant, respectively. More than a quarter (25.70%) of the respondents was current contraceptive users. The prevalence of HIV/AIDS was 1.21% (175). A total of 3,425 (23.63%) women were anaemic. The prevalence of mild, moderate and severe anaemia was 17.8% (2,584), 5% (730) and 0.8% (111), respectively. The median Hb concentration was 12.90 g/dl (interquartile range [IQR]: 11.7–13.90; Table 5).

**TABLE 5:** Characteristics of the women of reproductive age in Ethiopia, 2018: Data from 2016 Ethiopia Demographic and Health Survey (*n* = 14,489)

Variables	Frequency (*n*)	Percentage (%)
Individual level characters		
Age		
15–24	5,628	38.84
25–34	4,935	34.06
35–49	3,926	27.10
Women education		
No	7,005	48.35
Primary	5,092	35.14
Secondary and above	2,392	16.51
Marital status		
Living with husband	9,516	65.67
Not living with husband	4,973	34.33
Religion		
Protestant	3,411	23.55
Orthodox	6,259	43.20
Muslim	4,510	31.12
Other	309	2.12
Mass media exposure		
No	10,921	75.37
Yes	3,568	24.63
Alcohol consumption		
None	9,504	65.60
< once/week	3,045	21.01
At least once/week	1,635	11.28
> once/week	305	2.10
Chewing khat		
No	12,931	89.25
Yes	1,558	10.75
Smoking		
No	14,375	99.21
Yes	114	0.78
Maternity status		
NP‐NL	9,012	62.20
Pregnant	1,055	7.29
Lactating	4,422	30.52
Contraceptive use		
None	10,765	74.30
Pill/injectable/implants	3,368	23.24
IUD	214	1.48
Nonhormonal	142	0.98
Births in last 5 years		
No	7,374	50.89
One child	4,344	29.99
>one child	2,771	19.11
History of abortion		
No	1,447	96.98
Yes	442	3.05
Use of deworming drug		
No	13,984	96.51
Yes	505	3.49
Iron supplementation		
No	11,602	80.07
Yes	2,887	19.93
Nutritional counselling		
No	11,515	79.47
Yes	2,974	20.53
HIV status (*n* = 14,465)		
Negative	14,290	98.79
Positive	175	1.21
Household level characteristics		
Wealth index	`	
Poorest	2,603	17.97
Poorer	2,737	18.89
Middle	3,016	20.82
Rich	3,049	21.04
Richest	3,084	21.28
Family size		
≤2	1,207	8.33
3 and 4	3,992	27.55
≥5	9,290	64.12
Cooking fuel		
Solid	13,564	93.62
Clean	925	6.38
Toilet facility		
Improved	2,469	17.04
Unimproved	12,020	82.96
Community level characteristics		
Water source		
Unimproved	4,861	33.55
Improved	9,628	66.45
Place of residence		
Rural	11,412	78.77
Urban	3,077	21.23
Community women education		
Low	7,357	50.78
High	7,132	49.22
Community mass media exposure		
Low	7,213	49.79
High	7,276	50.21
Community poverty		
High	7,351	50.74
Low	7,138	49.26
Community unemployment		
High	7,343	50.68
Low	7,146	49.32

Abbreviations: IUD, intrauterine device; NP‐NL, neither pregnant nor lactating; other, Catholic, traditional or other religion.

### 3.1 Multivariable multilevel OLR result

#### 3.1.1 Random effects

Five random intercept models were fitted (Models 1, 2, 3, 4 and 5).

##### Model 1 (null model)

It is the intercept only model without independent variable. It provided the variance components of random effects which helped to determine variation between households and clusters to favour multilevel analysis over a standard ordered logistic regression.

This model showed that more than one‐third (VPC_(2 + 3)_ = 35.64%) of the total variation in anaemia was due to unobserved household and community level factors. About a quarter (VPC_(3)_ = 24.09%) and 11.56% (VPC_(2)_) of the variation was attributable to community and household level unobserved factors, respectively. This quantity of VPC_(2 + 3)_ was a suggestive of using multilevel model rather than single‐level model. Furthermore, the intrahousehold clustering (VPC = 11.6%) was a suggestive of using three‐level model rather than two‐level which ignores the household level variability (controlling for intrahousehold and intracluster variability). Models 2, 3 and 4 are models adjusted for individual, household and community level variables, respectively (Table 6).

**TABLE 6:** Random intercept variances and model fit statistics of three‐level mixed effect models

Random effects	Model 1	Model 2	Model 3	Model 4	Model 5
σ^2^(ν3)	1.23	0.68	0.90	0.42	0.36
σ^2^(ν2)	0.59	0.60	0.57	0.63	0.36
VPC_(2 + 3)_ (%)	35.64	28.15	30.90	24.29	18.00
VPC_(3)_ (%)	24.09	14.97	18.81	9.70	9.00
VPC_(2)_ (%)	11.56	13.12	11.90	14.50	9.00
PCV_3_ (%)		44.71	26.82	68.85	70.73
Model fit statistics					
AIC	21171.12	20675.28	21054.41	20766.5	20412.04

*Note*: σ^2^(ν3) and σ^2^(ν2) are community and household random intercept variances, respectively. VPC_(3)_, variance partition coefficient for cluster, VPC_(2 + 3)_, variance partition coefficient for household and cluster, VPC_(2)_, variance partition coefficient for household. PCV_3_, proportional change in cluster level variance; Model 1, model with no independent variable; Model 2, model adjusted for individual level variables; Model 3, model adjusted for household level variables; Model 4, model adjusted for community level variables; Model 5, model adjusted for individual, household and community level variables simultaneously.

Abbreviation: AIC, Akaike information criteria.

##### Model 5: Adjusted for individual, household and community level factors together

This model showed that 18% (VPC_(2 + 3)_), 9% (VPC_(3)_) and 9% (VPC_(2)_) of the unexplained variation in anaemia could be attributable to unobserved community and household level factors together, community level factors alone and household level factors alone, respectively. The PCV_3_ showed that 70.73% of the cluster level variation was explained by the variables in the model. This suggested that, after controlling for the variables in the model, there are still significant factors of anaemia at household and community levels which are not accounted for. Model 5 was found to be the best fit model for the data (Table 6).

#### 3.1.2 Factors associated with anaemia

Table 7 (Model 5) presents factors associated with anaemia among women. Except for the variable pregnancy, the same odds ratios were used and interpreted comparing higher versus lower level of anaemia (severe versus moderate and below, moderate and severe versus mild and none, mild and above versus none anaemia). This is because the proportional odds assumption was violated for only pregnancy.

**TABLE 7:** Factors associated with anaemia among women of reproductive age in Ethiopia, 2018: Data from 2016 EDHS (*n* = 14,489)

Variables	Model 2 AOR [95% CI]	Model 3 AOR [95% CI]	Model 4 AOR [95% CI]	Model 5 AOR [95% CI]
HIV status				
Negative	1			1
Positive	2.5 [1.85, 3.40]^***^			2.40 [1.76, 3.25]^***^
Maternity status				
NP‐NL	1			1
Pregnant	1.40 [1.20, 1.70]^***^			2.30 [1.82, 2.91]^***^
Lactating	1.0 [0.87, 1.15]			1.10 [0.96, 1.25]
Contraceptive use				
None	1			1
Pill/injectables/implants	0.5 3 [0.5, 0.70]^***^			0.67 [0.59, 0.77]^***^
IUD	1.20 [0.83, 1.80]			1.43 [0.98, 2.11]
Nonhormonal	1.10 [0.73, 1.5]			1.22 [0.81, 1.82]
Births in 5 years				
No	1			1
One child	1.37 [1.18, 0.60]^***^			1.20 [1.05, 1.40]^**^
>one child	1.75 [1.48, 2.10]^***^			1.40 [1.19, 1.71]^***^
Women education				
None	1			1
Primary	0.78 [0.60, 0.80]^***^			0.88 [0.79, 1.00]
Secondary and above	0.60 [0.59, 0.80]^***^			0.83 [0.71, 0.97]^*^
Marital status				
Living with husband	1.22 [1.05, 1.50]^*^			1.20 [1.04, 1.35]^*^
Not living with husband	1			1
Religion				
Protestant	1			1
Orthodox	0.73 [0.6, 0.9]^***^			0.73 [0.60, 0.88]^**^
Muslim	1.6 [1.3, 1.97]^***^			0.97 [0.81, 1.18]
Other	1.12 [0.76, 1.64]			1.07 [0.74, 1.43]
Wealth index				
Poorest		2.11 [1.75, 2.54]^***^		1.34 [1.2, 1.61]^**^
Poorer		1.57 [1.31, 1.87]^***^		1.29 [1.11, 1.56]^**^
Middle		1.38 [1.16, 1.64]^***^		1.25 [1.08, 1.49]^**^
Richer		1.14 [0.97, 1.34]		1.13 [0.90, 1.33]
Richest		1		1
Family size				
≤2		1		1
3 and 4		1.2 [1.01, 1.43]^*^		1.12 [0.94, 1.32]
≥5		1.4 [1.2, 1.6]^***^		1.24 [1.05, 1.47]^*^
Place of residence				
Rural			2 [1.6, 2.57]^***^	1.57 [1.28, 1.92]^***^
Urban			1	1
Region				
Tigray			1	1
Afar			3.39 [2.4, 4.8]^***^	2.09 [1.49, 2.9]^***^
Amhara			0.75 [0.6, 1.02]	0.74 [0.6, 1.0]
Oromia			1.51 [1.1, 2.05]^**^	1.07 [0.8, 1.44]
Somali			8.15 [5.8, 11.4]^***^	4 [2.9, 5.47]^***^
Benishangul Gumuz			0.84 [0.60, 1.27]	0.65 [0.47, 0.91]^*^
SNNPR			0.99 [0.72, 1.35]	0.77 [0.55, 1.04]
Gambela			1.67 [1.2, 2.34]^**^	1.15 [0.81, 1.61]
Harari			2.27 [1.56, 3.3]^***^	1.44 [1.01, 2.09]^*^
Addis Ababa			1.57 [1.09, 2.3]^*^	1.3 [0.92, 1.84]
Dire Dawa			2.81 [1.9, 4.07]^***^	1.93 [1.34, 2.74]^***^

*Note*: 1, reference category; other, Catholic and traditional religions; Model 5, partial proportional odds model; Models 2, 3 and 4 are proportional odds models.

Abbreviations: AOR, adjusted odds ratio; CI, confidence interval; EDHS, Ethiopia Demographic and Health Survey; IUD, intrauterine device; NP‐NL, neither pregnant nor lactating.

^*^ *P* value < 0.05.

^**^ *P* value < 0.01.

^***^ *P* value < 0.001.

HIV positive women were 2.4 times more likely to be at higher levels of anaemia as compared with HIV negative women. Pregnant women had 1.2 times higher odds of mild and above anaemia than neither pregnant nor lactating women (NP‐NL) when mild and above anaemia are compared with no anaemia. When moderate and above levels of anaemia are compared with mild and no anaemia levels, pregnant women had 2.3 times higher odds of moderate and above anaemia than NP‐NL women. Pregnant women had 1.87 times higher odds of severe anaemia than NP‐NL women when severe anaemia is compared with moderate and lower anaemia.

Current users of contraceptive pills, implants or injectable were 33% less likely to be at higher levels of anaemia as compared with nonusers of contraceptive. Women who gave birth to one and more than one child were 1.2 and 1.4 times more likely to be at higher levels of anaemia as compared with women with no birth, respectively. Women with secondary and above education were 17% less likely to be at higher levels of anaemia as compared with noneducated women. Relative to those not living with husbands, women living with husbands had 20% higher odds of having worse anaemia. Women in households with poorest, poorer and middle wealth index had 1.34 times greater odds of being at higher levels of anaemia as compared with the women of richest households, respectively. Women living with five or more family members had 1.24 times greater odds of being at higher levels of anaemia as compared with the women living with two or less family members.

The odds of having higher levels of anaemia were 1.57 times greater for rural women as compared with the urban women. The chances of having worse anaemia were 2.09, 4, 1.44 and 1.93 times greater for women living in Afar, Somali and Harari regions and Dire Dawa city, respectively, as compared with women living in Tigray region. Women living in Benishangul Gumuz region had 35% lesser odds of worse anaemia as compared with the women living in Tigray region (Table 7).

## 4 DISCUSSION

The main aim of this study was identifying multilevel (individual, household and community level) factors associated with anaemia among women in Ethiopia. Accordingly, the study found that anaemia was influenced by factors evolving not only at individual level but also at household and community (cluster) levels factors too. There was a sizable heterogeneity in a likelihood of anaemia across households and clusters. This heterogeneity implied that the entire variation in anaemia might not be fully explained by individual level factors alone.

HIV positive women had greater odds of being at higher levels of anaemia than HIV negative women. Similar association was observed in studies from Malawi (Adamu et al., 2017) and Ethiopia (Ejigu, Wencheko, & Berhane, 2018; Melku, Addis, Alem, & Enawgaw, 2014). This could be due to blood loss, decreased and ineffective RBC production and increased RBC destruction associated with comorbidities, medications and micronutrient deficiencies in HIV positive women (Volberding et al., 2004). This is in contrast to another study in Ethiopia (Bekele, Tilahun, & Mekuria, 2016) where HIV status was not associated with anaemia. This might be due to the possibility that HIV positive women in the later study might be in the early stage of the disease or properly screened and treated for anaemia, and they might had high adherence to antiretroviral drugs and other interventions.

Pregnant women had higher odds of anaemia than pregnant or lactating women. Furthermore, its effect was stronger for moderate and severe anaemia than for mild and above anaemia. Findings from Tanzania (Wilunda et al., 2013), Malawi (Adamu et al., 2017) and Ethiopia (Ejigu et al., 2018) also supported the observed association. The likely explanations to this association could be the increased risk of infections and obstetric complications during pregnancy that leads to blood loss and under nutrition (Balarajan et al., 2011; Ebuy, Alemayehu, Mitiku, & Goba, 2017). Use of pills, implants or injectable was negatively associated with anaemia. This is consistent to the findings from Tanzania (Wilunda et al., 2013), Bangladesh (Kamruzzaman et al., 2015) and Ethiopia (Asres, Yemane, & Gedefaw, 2014; Lakew, Biadgilign, & Haile, 2015). The noncontraceptive benefits of the hormonal contraceptives like iron content of the pills, prevention of the heavy menstrual bleeding and regulating menses could explain the low prevalence of anaemia among contraceptive users (Brant, Ye, Teng, & Lotke, 2017).

Women with one or more births in the last 5 years before survey had greater odds of being at higher levels of anaemia than women with no births in the last 5 years. This finding is in agreement with the results from studies in India (Perumal, 2014) and Ethiopia (Bekele et al., 2016). This could be due to the possibility that narrow birth interval hinders the restoration of iron and other micronutrient stores between pregnancies. Also, women with birth history could have history of obstetric complications such as postpartum haemorrhage and infections which directly expose them to anaemia (Balarajan et al., 2011; Ebuy et al., 2017; Federal Ministry of Health [FMOH], 2008).

Women with secondary and above education were less likely to be at higher levels of anaemia as compared with noneducated women. This is in agreement to the findings from studies in China (Ma et al., 2017), Bangladesh (Kamruzzaman et al., 2015) and Ethiopia (Ejigu et al., 2018). There is evidence that women with secondary and higher education have higher health seeking behaviour and service utilization than noneducated women so that they get preventive and curative services for conditions that contribute for anaemia (Bobo, Yesuf, & Woldie, 2017; Wonde & Tadele, 2015). However, this finding is in conflict with another study in Ethiopia where education was not associated with anaemia (Kefiyalew, Zemene, Asres, & Gedefaw, 2014). This might be due to the reason that the latter study has controlled for factors which might be more proximal factors of anaemia than education.

Wealth index of the household was positively associated with anaemia in women. This is supported by the findings in China (Siddiqui et al., 2017) and Bangladesh (Kamruzzaman et al., 2015). Inability to afford micronutrient‐rich food which is expensive in Ethiopia and lack of priority to women in poor households could make them more vulnerable to anaemia than women of richest households. There is also evidence that poor households are poor in seeking and timing health care so that they might not be treated for diseases that may result in anaemia (Bobo et al., 2017; FMOH, 2008; Mebratie et al., 2014). This finding disagrees with the finding in another study done in Ethiopia (Ejigu et al., 2018) where wealth index was not associated with anaemia. This might be due to the increase in the prices of foods and health services over time that widens the difference between richest and poorest in terms of affording foods and health services in Ethiopia.

Women who were living with five or more family members had greater odds of higher levels of anaemia as compared with those living with two or less family members. This is in agreement with findings in Ethiopia (Bekele et al., 2016). This could be due to the possibility that large family size might results in household food insecurity which compromises women's access to balanced diet. In addition, traditionally women might be expected to serve their family first and eat the leftover if any at the end.

Rural women had greater odds of higher levels of anaemia as compared with urban women. The same evidence was reported in studies from Ethiopia (Ebuy et al., 2017; Ejigu et al., 2018; Kefiyalew et al., 2014). Due to poor health seeking behaviour and service utilization associated with inadequate infrastructure, rural women might not get care for maternal services and disease which may contribute to anaemia (Begashaw, Tessema, & Gesesew, 2016; Bobo et al., 2017). This finding disagrees with a study conducted in Malawi (Adamu et al., 2017) in which place of residence was not related to anaemia. The difference in health service utilization and food access and sociocultural differences between the countries might explain the disagreement between studies. Unlike Ethiopia, national estimates of the maternal health service utilization were higher, and there was little difference between urban and rural women in terms of maternal health service utilization in Malawi (Malawi, 2015).

Women who were living in Somali, Afar and Harari regions and Dire Dawa city had greater odds of higher levels of anaemia as compared with women living in Tigray region. This finding is in line to the findings from other studies in China (Ma et al., 2017), Tanzania (Wilunda et al., 2013) and Ethiopia (Alemu & Umeta, 2015). Differences in dietary preferences and disease burden, inequalities in access to health care across the regions and difference in societal beliefs, cultural practices towards the care for women and climatic conditions might have contributed to the observed difference in odds of anaemia. Restrictive dietary behaviour was positively associated with anaemia in Somali region where women are expected to reduce the size and frequency of meal during pregnancy (Kedir, Berhane, & Worku, 2013). Also, a study in Afar region revealed that eating large amount of any food, meat and solid food are tabooed for pregnant women which may result them to malnutrition (Hadush, Birhanu, Chaka, & Gebreyesus, 2017). The lowest utilization of maternal health services in Afar and Somali (Bobo et al., 2017) and recurrent drought‐triggered food insecurity might have contributed for the higher prevalence of anaemia in these regions (FMOH, 2008).

Despite the existing prevention strategies, anaemia remains a moderate public health problem in WRA. This is a suggestive of significant contribution of other causative factors than those addressed by current strategies (haemoglobinopathies and vitamin B deficiencies) and allocating more attention and resources to determine aetiology of anaemia. Determining aetiologic indicators of anaemia like iron status (ferritin), inflammatory markers and C‐reactive protein (CRP), malaria status, genetic blood disorders and haemoglobinopathies in population‐based survey would be important to implement context‐specific interventions.

Being a moderate public health problem also suggests the coverage and consistency issue of the current intervention methods need to be considered. Monitoring and evaluation of programmes is inadequate in many countries. Anaemia prevention programmes have challenges which include poor attendance at antenatal clinics or insufficient emphasis on behavioural aspects of taking supplements on a regular basis and consuming diverse diets which have limited their effectiveness (World Health Organization, 2016). Revision of existing programmes would be important to provide evidence on challenges and methods of improving their effectiveness.

### 4.1 Limitation of the study

Cross‐sectional nature of the study which cannot show the temporal relationship between risk factors and anaemia. Use of secondary data and inability to include variables like hookworm infection, malaria, schistosomiasis, menorrhagia, chronic diseases, dietary intake, cultural taboos and body mass index might made difficult to identify the independent effects of the considered variables. Recall bias might be introduced during measuring some events in the past.

## 5 CONCLUSIONS

Anaemia was found to be influenced by factors evolving at individual, household and community levels. At individual level, HIV infection, pregnancy, increased number of births and living with husband were positively associated with anaemia. However, secondary and above education and use of contraceptive pills, implants or injectable were negatively associated with anaemia. At household level, living in households with large family members and with poorest, poorer and middle wealth index was positively associated with anaemia. Living in rural areas, in Afar, Somali and Harari regions and Dire Dawa city was positively associated with anaemia. There was a sizable heterogeneity in likelihood of anaemia across households and clusters which is unaccounted for by the predictor variables.

Daily iron supplementation during pregnancy is recommended as part of the standard care by WHO (World Health Organization, 2012). A daily iron supplementation to women during pregnancy was found to reduce anaemia at term by 70% (Bhutta et al., 2013; Peña‐Rosas, De‐Regil, Garcia‐Casal, & Dowswell, 2015). Countries like Indonesia and Sweden have effectively controlled anaemia in pregnant women (Suharno, Karyadi, West, & Hautvast, 1993; World Health Organization, 2001). The Ethiopia FMoH should strengthen anaemia prevention and control programmes for WRA living with HIV/AIDS and during pregnancy. To tackle the anaemia among poor, household poverty reduction and social protection services should be strengthened and integrated in anaemia prevention and controlling programmes in women. Information on dietary intake of women and indicators of infections need to be incorporated during DHS, and further study is needed to determine additional factors.

## CONFLICTS OF INTEREST

The authors declare that they have no conflicts of interest.

## CONTRIBUTIONS

LLT conceived and designed the research and wrote the first draft of the paper. AM, AB and DE reviewed the paper. LLT, AS, MG and RA analysed and interpreted the result. All authors read and edited the first draft of the paper.
